# Similarity-Reduced Diversities: the Effective Entropy and the Reduced Entropy

**DOI:** 10.1007/s00357-021-09395-4

**Published:** 2021-09-08

**Authors:** François Bavaud

**Affiliations:** Department of Language and Information Sciences, Institute of Geography and Sustainability University of Lausanne, Lausanne, Switzerland

**Keywords:** Similarity-reduced diversity, Confusion matrix, Rate distortion function, Phase transitions, Rao quadratic entropy

## Abstract

The paper presents and analyzes the properties of a new diversity index, the effective entropy, which lowers Shannon entropy by taking into account the presence of similarities between items. Similarities decrease exponentially with the item dissimilarities, with a freely adjustable discriminability parameter controlling various diversity regimes separated by phase transitions. Effective entropies are determined iteratively, and turn out to be concave and subadditive, in contrast to the reduced entropy, proposed in Ecology for similar purposes. Two data sets are used to illustrate the formalism, and underline the role played by the dissimilarity types.

## Introduction

Evaluating the variety or diversity of a collection of items occurring with unequal frequencies is a much explored domain of data analysis, with well-known proposals such as the logarithmic Shannon entropy, or Rényi or Tsallis power generalizations (see, e.g., Masi, 2005; Jost, 2006). In those *plain* or *naive* approaches, items are implicitly meant as completely dissimilar from each other. Yet, examples abound in which some pairs of items are *similar* to some extent, thus arguably reducing the corresponding naive diversities. The quest for formally sound and fruitfully interpretable *similarity-dependent* measures of diversity has been explicitly formulated and investigated in the ecological literature (see, e.g., Champely & Chessel, 2002; Pavoine et al., 2005; Leinster & Cobbold, 2012; Chiu et al., 2014; Marcon, 2016and references therein).

This paper proposes an original similarity-dependent logarithmic measure of relative diversity (6), we shall refer to as the *effective entropy*. It depends upon the relative frequencies and dissimilarities between items, as well as on a freely adjustable parameter controlling for the *discriminability* between similar items. Its construction can be motivated by a simple perceptual rationale: if two items are similar enough, and if the discriminability parameter is low enough, then one item can wrongly be perceived as another, hence lowering the diversity of the item collection. Further elaborating on this idea leads to a statistical mechanical framework (minimization of the free energy) whose associated formalism turns out to overlap in large parts with the *rate distortion setup* in information theory (e.g., Cover & Thomas, 2006) or the *regularized optimal transportation* (e.g., Cuturi, 2013) — with a completely different motivation and purpose.

The resulting effective entropy can be computed iteratively in general (Section 2, Theorems 1 and 2), sometimes in a single step for particular cases. One of the salient features of the framework is the apparition of *phase transitions* when lowering the discriminability parameter, from the *low-temperature regime* where all the items are perceived, at least partially, to the *high-temperature regime*, where only the *dominating items* persist (Section 3, Theorems 3, 4 and 5).

Two examples (copper concentration data, Section 3.1; world cities, Section 4.1) illustrate the formalism and its versatility, as well as the influence of the nature (city-block, Euclidean, ultrametric) of the item dissimilarities (Theorems 6 and 7). *Similarities* can also be considered directly, as far they are non-negative, symmetric, and endowed with a unit dominating diagonal.

The effective entropy (6) turns out to provide an (apparently close) upper bound to another logarithmic measure of diversity (??) considered in Ecology and computable in a single step, referred in this paper to as the *reduced entropy*. The presence of entirely similar items with distinct labels should not increase the diversity — a property automatically satisfied by the effective and the reduced entropies, in contrast to Shannon entropy. Also, the former satisfy the so-called monotonicity and modularity properties (Theorem 8). Both take on their maximum values for non-uniform item distributions in general (Theorem 10), and converge to Shannon entropy in the *naive limit* of identity similarities, as expected.

Yet, the *concavity property*, asserting that the diversity of the whole should not be lower than the average diversity of its parts, is in general satisfied by the effective entropy only (Theorem 9). Also, like Shannon entropy, the joint effective entropy is maximum for independent distributions (Theorem 11), a property violated again in general for the reduced entropy. Remedying the formal defects of the latter provided the initial motivation of the present study.

## The Formalism

Basic ingredients consist of *n* items, objects or species, with relative frequencies *f*_*i*_ > 0 normalized to ${\sum }_{i=1}^{n} f_{i}=1$. In addition, the differences between items are specified by a finite *n* × *n* matrix of *dissimilarities* ***D*** = (*d*_*ij*_) obeying

1a $$ \begin{array}{@{}rcl@{}} d_{ij}&\ge&0 \end{array} $$

1b $$ \begin{array}{@{}rcl@{}} d_{ij}&=&d_{ji} \end{array} $$

1c $$ \begin{array}{@{}rcl@{}} d_{ii}&=&0 \end{array} $$

1d $$ \begin{array}{@{}rcl@{}} d_{ij}&=&0  \Rightarrow  d_{ik}=d_{jk}, \end{array} $$

where (1d) defines an *even* or *semi-proper* dissimilarity (see, e.g., Critchley & Fichet, 1994).

### Transition Matrices and Percept Weights

Let *z*_*ij*_ denote the probability that item *i* is perceived or received as item *j*. According to the context, the pair *i*-*j* can be referred to as the stimulus-percept, stimulus-response, origin-destination, input-output, symbol-reproduction, or source-estimate pair. By construction, *z*_*ij*_ ≥ 0 and $z_{i\bullet }={\sum }_{j} z_{ij}=1$ (where “∙” denotes summation over the replaced index). The set of all such memberships or *transition matrices* $\boldsymbol {Z}=(z_{ij})\in \mathbb {R}^{n\times n}$ will be denoted as ${\mathcal Z}$.

The *weight of percept* *j* is obtained as

2 $$  \rho_{j}=\sum\limits_{i} f_{i} z_{ij}\ge0 $$

and obeys ***ρ***_∙_ = 1 by construction.

### Energy, Mutual Information, and Free Energy

The average stimulus-percept dissimilarity, cost or *energy* is

3 $$  U[\boldsymbol{Z}]=\sum\limits_{i,j}^{n} f_{i} z_{ij}d_{ij}\ge0, $$

whose minimum is ***Z*** = ***I***_*n*_ = (*δ*_*ij*_), the identity matrix (no stimulus-percept confusion). This minimum is unique if ***D*** is *proper*, that is if *d*_*ij*_ > 0 for *i*≠*j*.

By contrast, the confusion is maximum when the percept is independent of the stimulus, that is when the joint stimulus-percept distribution *f*_*i*_*z*_*ij*_ is equal to the corresponding independent distribution *f*_*i*_*ρ*_*j*_, or equivalently when *z*_*ij*_ = *ρ*_*j*_ for the distribution ***ρ*** in (2). Such an independent transition minimizes the stimulus-percept *mutual information*, which reads here as

4 $$  K[\boldsymbol{Z}]=\sum\limits_{i,j}^{n} f_{i} z_{ij}\ln \frac{z_{ij}}{\rho_{j}} \ge0. $$

The above antagonist tendencies can be combined in the (dimensionless) *free energy*

5 $$  F[\boldsymbol{Z}]=\beta U[\boldsymbol{Z}]+K[\boldsymbol{Z}], $$

where *β* > 0 is a free *discriminability* parameter, known in statistical mechanics as the *inverse temperatureβ* = 1/*T*. In the *low-temperature limit* $\beta \to \infty $, only the energy term contributes and the optimal transition matrix ***Z*** tends to ***I***_*n*_ (perfect discrimination). In the *high-temperature limitβ* → 0, only the entropy term contributes and $\boldsymbol {Z}\to \boldsymbol {1}_{n}\boldsymbol {\rho }^{\prime }$ (complete confusion).

Note that in physics, the free energy is generally defined as *U* + *β*^− 1^*K* instead of *F* in (5). The latter choice, without incidence on the form of the optimal transition matrix ***Z***, has the advantage of making the effective entropy (defined below) dimensionless, and directly comparable to Shannon entropy. Also, ***D***, *U* and *β*^− 1^ have the dimensions of an energy; they can be made dimensionless by a proper rescaling of ***D*** (see Section 3.1).

### Effective Entropy *E* and Reduced Entropy *R*

The *effective entropy*, whose study constitutes the main scope of the paper, is defined as

6 $$  E\equiv E(\boldsymbol{f},\boldsymbol{D},\beta)=\min_{\boldsymbol{Z}\in {\mathcal Z}}F[\boldsymbol{Z}]. $$

The optimal transition ***Z*** minimizing *F*[***Z***] is determined by the non-linear equation (see Theorem 1 below)

7 $$  z_{ij}=\frac{s_{ij}\rho_{j}}{\tau_{i}}, \qquad\qquad \text{where}\qquad\quad s_{ij}=e^{-\beta d_{ij}}, $$

8 $$  \quad \rho_{j}{\scriptstyle[\boldsymbol{Z}]}=\sum\limits_{i} f_{i} z_{ij}\qquad \qquad \text{and}\qquad\quad \tau_{i}{\scriptstyle[\boldsymbol{Z}]}=\sum\limits_{j} s_{ij} \rho_{j}. $$

The components of the *n* × *n* *similarity matrix* ***S*** = (*s*_*ij*_) in (7) obey

9a $$ \begin{array}{@{}rcl@{}} s_{ij}&\ge&0 \end{array} $$

9b $$ \begin{array}{@{}rcl@{}} s_{ij}&=&s_{ji} \end{array} $$

9c $$ \begin{array}{@{}rcl@{}} s_{ii}&=&1 \end{array} $$

9d $$ \begin{array}{@{}rcl@{}} s_{ij}&=&1  \Rightarrow  s_{ik}=s_{jk}. \end{array} $$

Similarities (??) are related to dissimilarities as $\boldsymbol {S}=\exp (-\beta \boldsymbol {D})$, to be understood as the *componentwise* or *Hadamard exponential*. In terms of similarities, the free energy (5) reads, by direct substitution, as

10 $$  F[\boldsymbol{Z}]=\sum\limits_{i,j}^{n} f_{i} z_{ij}\ln \frac{z_{ij}}{s_{ij}\rho_{j}} \ge0. $$

Let $\kappa (\boldsymbol {\rho }||\boldsymbol {f})={\sum }_{j} \rho _{j} \ln (\rho _{j}/f_{j})\ge 0$ denote the Kullback-Leibler divergence between percept and stimulus weights, and consider the functional

11 $$  G[\boldsymbol{Z}]=F[\boldsymbol{Z}]+\kappa(\boldsymbol{\rho}||\boldsymbol{f})=\sum\limits_{i,j}^{n} f_{i} z_{ij}\ln \frac{z_{ij}}{s_{ij}f_{j}} \ge0. $$

Its minimizer ***Z***^0^, and minimum value *R* = *G*[***Z***^0^], we shall refer to as the *reduced entropy*, are readily found (in a single step) to be

12a $$ \begin{array}{@{}rcl@{}} z^{0}_{ij}&=&\frac{s_{ij}f_{j}}{b_{i}},\qquad\text{where}\quad b_{i}=\sum\limits_{j} s_{ij}f_{j}, \text{and} \end{array} $$

12b $$ \begin{array}{@{}rcl@{}} R&=&\min_{\boldsymbol{Z}\in {\mathcal Z}}G[\boldsymbol{Z}]=-\sum\limits_{i} f_{i} \ln b_{i}\qquad\text{(reduced entropy}). \end{array} $$

Quantity *b*_*i*_ in (??) is the *banality* of item *i*, measuring its average similarity to other items (Marcon, 2016), proposed by Leinster and Cobbold (2012), as well as by Ricotta and Szeidl (2006) in the variant $s_{ij}=1-d_{ij}/d_{\max \limits }$. By construction,

13 $$  0\le E\le R\le H,\quad\text{where}\quad H=-\sum\limits_{i} f_{i} \ln f_{i}\quad\text{(Shannon entropy)}. $$

The lower bound for *R* follows from (11), and the upper bound for *R* from *f*_*i*_ ≤ *b*_*i*_ ≤ 1. Also, substituting (7) in (10) yields the expression

14 $$  E=-\sum\limits_{i} f_{i} \ln \tau_{i}. $$

The normalization factor *τ*_*i*_ in (7) and (8) has a form similar to *b*_*i*_ in (??): they both measure the average similarity, where the average is taken on ***f*** for *b*_*i*_, but on ***ρ*** for *τ*_*i*_. One can refer to *b*_*i*_ as the source or *stimulus banality*, and to *τ*_*i*_ as the outcome or *percept banality*.

### High- and Low-Temperature Similarities: Aggregation of Equivalent Items

In the high-temperature limit *β* → 0, ***S*** →***J***_*n*_ (the *n* × *n* unit matrix filled with ones), and thus ***b*** →***1***_*n*_ (the unit vector) and finally *E*,*R* → 0.

In the low-temperature limit $\beta \to \infty $, *s*_*ij*_ → 0 for *i*≠*j*, unless *d*_*ij*_ = 0: recall from (1d) that ***D*** is supposed to be semi-proper only, which implies that the relation $i\sim j$ ⇔ *d*_*ij*_ = 0 is an equivalence relation. As a result, $\lim _{\beta \to \infty }s_{ij}=1$ if *i* and *j* belong to the same equivalence class *C*, and $\lim _{\beta \to \infty }s_{ij}=0$ otherwise. Hence, $\boldsymbol {S}=\bigoplus _{C} \boldsymbol {J}_{C}$ (direct sum of matrices) is block diagonal, and

15 $$  \lim_{\beta\to\infty}R=H_{\text{\scriptsize agg}}=-\sum\limits_{C} F_{C}\ln F_{C}, \qquad\text{where}\quad F_{C}=\sum\limits_{i\in C} f_{i}. $$

See the Appendix for a proof of (15). In other terms, in the low-temperature limit, the reduced entropy *automatically aggregates* equivalent items (i.e., whose dissimilarity is zero), yielding a corresponding entropy *H*_agg_ ≤ *H*. The same limit holds for the effective entropy.

Similar considerations hold for the effective and reduced entropies at any temperature: items equivalent in the sense *d*_*ij*_ = 0 can be first aggregated into equivalence classes, yielding *proper dissimilarities* between classes, on which the above formalism can then be applied. From now on, we consider proper dissimilarities only, i.e., such that *d*_*ij*_ > 0 for *i*≠*j*.

### Behavior and Computation of the Optimal Solution

Depending on *β*, the percept weights *ρ*_*j*_ may be positive or zero, a circumstance causing the phase transitions observed in the case studies below. The *effective variety*

16 $$  v_{\text{\scriptsize eff}}=\sum\limits_{j=1}^{n} I(\rho_{j}>0) $$

counts the number of detected percepts, and ranges from 1 to *n*.

Let 〈*n*〉 = {1,…,*n*} denote the collection of items, and for $A\subseteq \langle n \rangle $, let ${\mathcal Z}_{A}$ be the set of transitions whose non-zero percept weights are exactly those of *the set of occurring perceptsA*, that is *ρ*_*j*_ > 0 for *j* ∈ *A*, and *ρ*_*j*_ = 0 for *j*∉*A*. Equivalently, *z*_∙*j*_ > 0 for *j* ∈ *A*, and *z*_∙*j*_ = 0 for *j*∉*A*.

#### **Theorem 1** (first-order condition)

Define the *sub-indicator*

17 $$  c_{j}=\sum\limits_{i} \frac{f_{i}s_{ij}}{\tau_{i}}. $$

A necessary and sufficient condition for ***Z*** to be the minimizer of *F*[***Z***] is the first-order condition

18 $$  z_{ij}=\frac{s_{ij}\rho_{j}}{\tau_{i}}\quad\text{where}  \tau_{i}=\sum\limits_{j} \rho_{j} s_{ij}\qquad \quad \text{and}\qquad c_{j}\le 1\quad \text{when} \rho_{j}=0. $$

Also, *c*_*j*_ = 1 when *ρ*_*j*_ > 0.

In particular, the first identity in (18) is necessary and sufficient when all *ρ*_*j*_ > 0. Also, *c*_*j*_*ρ*_*j*_ = *ρ*_*j*_. Identity *c*_*j*_ = 1 is a necessary condition for the apparition of percept *j*, whence the name “sub-indicator”.

#### **Theorem 2** (iterative solution)

The series of iterations ***Z***^(0)^ →***ρ***^(0)^ →***Z***^(1)^ →***ρ***^(1)^ →⋯ with

19 $$  \rho_{j}^{(t)}=\sum\limits_{i} f_{i} z_{ij}^{(t)}\qquad\qquad z_{ij}^{(t+1)}=\frac{s_{ij} \rho_{j}^{(t)}}{\tau_{i}^{(t)}} \qquad\text{where}\quad \tau_{i}^{(t)}=\sum\limits_{j} s_{ij} \rho_{j}^{(t)} $$

converge towards the unique minimizer (7) of *F*[***Z***], provided $\boldsymbol {Z}^{(0)}\in {\mathcal Z}_{\langle n \rangle }$, the set of transitions with strictly positive row margins.

## Temperature Regimes and Illustrations

### Example A: Copper Concentration Data

The R dataset chem{MASS} consists of 24 univariate copper concentrations *x*_*i*_, on which dissimilarities are defined as *d*_*ij*_ = |*x*_*i*_ − *x*_*j*_|. The values 2.20 2.20 2.40 2.40 2.50 2.70 2.80 2.90 3.03 3.03 3.10 3.37 3.40 3.40 3.40 3.50 3.60 3.70 3.70 3.70 3.70 3.77 5.28 28.95 contain ties occurring twice (*x* = 2.20, 2.40, 3.03), three times (*x* = 3.40) and four times (*x* = 3.70). They must be preliminarily aggregated to define a proper dissimilarity (Section 2.4), resulting in a sample of *n* = 16 observations with non-uniform weights ***f***, namely with values 1/24 for the unique values, 2/24 for values with two ties in the original dataset, 3/24 for values with three ties, and 4/24 for values with four ties.

Temperatures and dissimilarities always appear in combination *βd*_*ij*_. For comparison sake, the temperature scale will, here and in the sequel, be fixed by further dividing the dissimilarities by the quantity

20 $$  {{\varDelta}}=\frac{1}{2}\sum\limits_{ij}f_{i}f_{j} d_{ij} $$

which corresponds to the *inertia* of the configuration when ***D*** is squared Euclidean, and is referred to as *Rao quadratic entropy* in Ecology (see, e.g., Champely & Chessel, 2002; Pavoine et al., 2005 and references therein). Equivalently, the dissimilarity scale is normalized to *Δ* = 1.

**Fig. 1:**
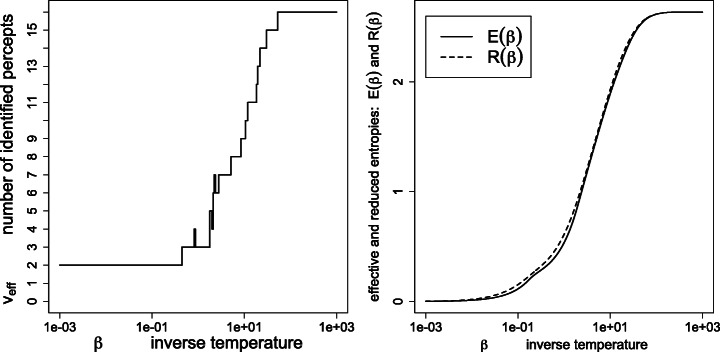
Left: effective variety *v*_eff_; right: effective entropy *E*(*β*) and reduced entropy *R*(*β*), as a function of the inverse temperature *β*

**Fig. 2:**
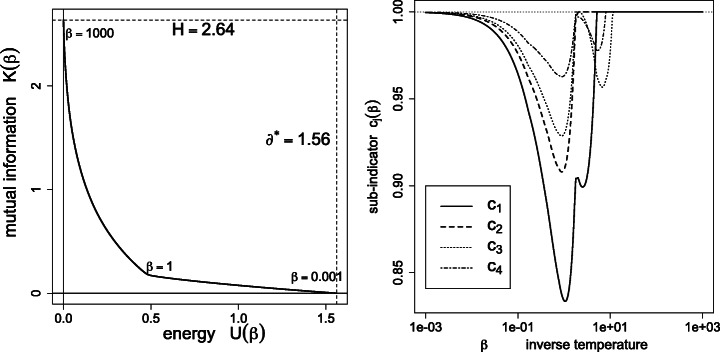
Left: rate distortion function, plotting the mutual information *K*(*β*) as a function of the energy *U*(*β*). Right: plot of the sub-indicators *c*_*j*_(*β*) (17) for *j* = 1,2,3,4

**Fig. 3:**
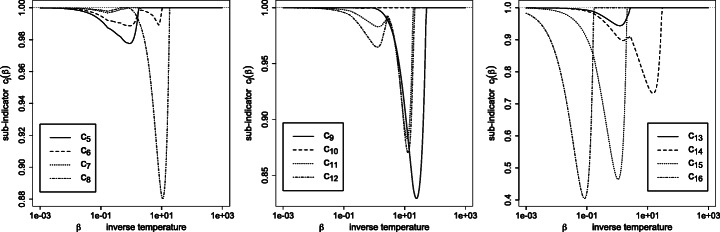
Plot of the sub-indicators *c*_*j*_(*β*) (17) for *j* = 5,6,7,8 (left), *j* = 9,10,11,12 (middle) and *j* = 13,14,15,16 (right)

Figure 1 depicts the effective variety (16), effective entropy (6) and reduced entropy (12b) as a function of the inverse temperature. As expected, the number of detected percepts *tends* to decrease with the temperature, yet with possible monotonicity violations (disappearances followed by re-emergence of percepts *ρ*_*j*_ > 0, as detailed in Figs. 2 and 3). Also, the effective entropy appears closely approximated by the more directly computable reduced entropy.

The left panel of Fig. 2 depicts the *rate distortion function* of information theory. In this framework (Cover & Thomas, 2006, chapter 10), source *i* is sent through a communication channel, and decoded as *j*. The mutual information *K* or *channel rate* between sources and their reconstruction is a measure of their dependence, reaching its maximum *H* (for fixed *f* ) for the perfect transmission ***Z*** = ***I***, in which case the *distortion* *U* is zero (Section 3.3). Conversely, a zero-rate or random channel (*K* = 0) generates a distortion of at least *U* = *∂*^⋆^ (Section 3.4).

The right panels of Figs. 2 and 3 depict the sub-indicators *c*_*j*_(*β*) (17) for *j* = 1,…,16. By construction, the effective variety is bounded above by the number of sub-indicators equal to one. Actually, the two quantities happen to numerically *coincide* in this case study, in the sense ${\sum }_{j=1}^{n} I(\rho _{j}>10^{-5})={\sum }_{j=1}^{n} I(c_{j}>{\small 1-10^{-5}})$. Said otherwise, *ρ*_*j*_ > 0 iff *c*_*j*_ = 1, and the sub-indicator *c*_*j*_ turns out to behave here as an indicator.

### High- and Low-Temperature Regimes

The previous sections suggest the existence of two critical temperatures *β*_*H*_ and *β*_*L*_, with $0<\beta _{H}<\beta _{L}<\infty $, such that

1. for *β* > *β*_*L*_, all *ρ*_*j*_ > 0 (that is $\boldsymbol {Z}\in {\mathcal Z}_{\langle n \rangle }$) : *low-temperature phase* or *regime*
2. for *β* < *β*_*H*_, *ρ*_*j*_ > 0 iff *j* belongs to some subset *A*^⋆^ ⊂〈*n*〉, the *dominating set*: *high-temperature regime*
3. for *β*_*H*_ < *β* < *β*_*L*_, the set *A* of occurring percepts with *ρ*_*j*_ > 0 varies, generally non monotonically with *β*, from *A*^⋆^ to 〈*n*〉: *medium-temperature regime*.

### Low-Temperature Regime

For $\beta \to \infty $, ***Z*** = ***I*** and *ρ*_*j*_ → *f*_*j*_ > 0. By continuity, for *β* large enough, all sub-indicators (17) are equal to one, and the inverse similarity matrix, noted ***S***^− 1^ = (*s*^*ij*^), exists (recall $\lim _{\beta \to \infty }\boldsymbol {S}=\boldsymbol {I}$ for ***D*** proper).

#### **Theorem 3** (low-temperature regime)

Assume that ***S***^− 1^ = (*s*^*ij*^) exists, and define the (possibly non positive) *signed weights* by

21 $$  r_{j}:=\sum\limits_{i=1}^{n}f_{i}\frac{s^{ij}}{s^{i\bullet}} . $$

Then all percepts emerge (that is *ρ*_*j*_ > 0 for all *j*) iff *r*_*j*_ > 0. In this case, the optimal solution (7), (8) is given by

22 $$  \rho_{j}=r_{j}  ,  \tau_{i}=\frac{f_{i}}{s^{i\bullet}}  ,  z_{ij}=\frac{s^{i\bullet}s_{ij}r_{j}}{f_{i}}\quad\text{and}\quad E=H+\sum\limits_{i} f_{i} \ln s^{i\bullet}(\beta). $$

Note that ${\sum }_{j}r_{j}=1$ always holds, whence the name signed weights. Thus the condition $\min \limits _{j}r_{j}\ge 0$, characterizing the low-temperature regime, also implies $\max \limits _{j}r_{j}~\le ~1$; see the left panels of Figs. 4, 7, 8, 9, 10, 11, 12, 13 and 14.

The invertibility of ***S*** for all *β* > 0 holds for a large class of dissimilarities, namely squared Euclidean proper dissimilarities (Theorem 6b). Yet, the above theorem does not rule out the possible coexistence of all percepts together with a non-invertible ***S***, although such a case has not been met in our investigations.

### High-Temperature Regime

For *β* = 0, the free energy consists of *K*[***Z***] only, which is minimized (and equal to zero) by the independent transition *z*_*ij*_ = *ρ*_*j*_, where *ρ* is an arbitrary distribution. Let us introduce the definitions

23a $$ \begin{array}{@{}rcl@{}} \partial_{j}&=&\sum\limits_{i}f_{i} d_{ij},\qquad\qquad \bar{\partial}=\sum\limits_{j} f_{j} \partial_{j}=2{{\varDelta}}, \end{array} $$

23b $$ \begin{array}{@{}rcl@{}} \partial^{\star}&=&\min\limits_{j} \partial_{j}\qquad\text{and}\qquad A^{\star}=\{j | \partial_{j} =\partial^{\star} \}, \end{array} $$

appearing in the study of the high-temperature regime. The *eccentricity∂*_*j*_ measures the (weighted) *average dissimilarity* between item *j* and the other items in the configuration (***f***,***D***), and obeys *∂*_*j*_ = *d*_*j****f***_ + *Δ* when ***D*** is squared Euclidean, where *d*_*j****f***_ is the dissimilarity between item *j* and the barycenter of the configuration (Huygens principle). The average eccentricity $\bar {\partial }$ is twice the Rao entropy (20), and is equal to 2 under the normalization *Δ* = 1. *Medoids* are items with minimal eccentricity *∂*^⋆^. The collection of medoids constitutes the *dominating setA*^⋆^.

**Fig. 4:**
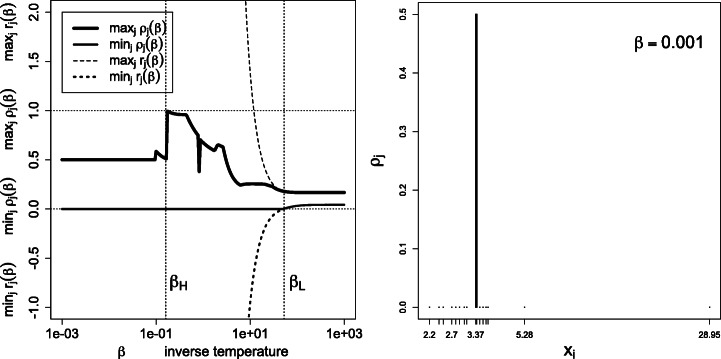
Left: the low-temperature regime *β* > *β*_*L*_ is characterized by $\min \limits _{j} r_{j}\ge 0$, and hence *r*_*j*_ = *ρ*_*j*_ (Theorem 3). Right: histogram of *ρ*_*j*_ in the high-temperature limit: the mass is evenly concentrated on the dominating states *A*^⋆^ = {9, 10}, that is *ρ*_9_ = *ρ*_10_ = 0.5

In the copper concentration data, the dominating set *A*^⋆^ contains the minimizers of the median average deviation $\partial _{j} ={\sum }_{i} f_{i} |x_{i}-x_{j}|$, and consists of the two observations *j* = 9 and *j* = 10 (see Fig. 4). In the world cities dataset with geodesic distances (Section 4.1), *A*^⋆^ consists of Berlin only.

For *β* → 0, the energy becomes $U[\boldsymbol {Z}]={\sum }_{j} \rho _{j}\partial _{j}$, which is minimum iff the non-zero components or *support* supp(***ρ***) of ***ρ*** belong to the dominating set *A*^⋆^. This dominating set persists in the high-temperature regime 0 < *β* ≪ 1:

#### **Theorem 4** (high-temperature regime)

For 0 < *β* ≪ 1, the free energy is minimum for

24 $$  z_{ij}=\frac{s_{ij}\rho_{j}}{{\sum}_{k\in A^{\star}}s_{ik}\rho_{k}}\qquad \text{\small where}\qquad \texttt{supp}(\boldsymbol{\rho})\subset A^{\star}. $$

Also, the effective and relative entropies obey

25 $$  E(\beta)=\beta \partial^{\star} +0(\beta^{2})\qquad\quad\text{and}\quad\qquad R(\beta)=\beta \bar{\partial}+0(\beta^{2}). $$

The dominating set is stable in the sense *c*_*j*_(*β*= 0) = 1 for all *j*, with derivatives $c_{j}^{\prime }{\scriptstyle (\beta =0)}=0$ for *j* ∈ *A*^⋆^ and $c_{j}^{\prime }{\scriptstyle (\beta =0)}<0$ for *j*∉*A*^⋆^, which implies (Theorem 1) *ρ*_*j*_(*β*) = 0 for 0 < *β* ≪ 1 and *j*∉*A*^⋆^.

Inequality $\partial ^{\star }\le \bar {\partial }$ reflects the general relation *E*(*β*) ≤ *R*(*β*). Also, when *A*^⋆^ contains a single element, the relation *E*(*β*) = *β∂*^⋆^ is exact in the high-temperature regime.

### Medium-Temperature Regime

**Fig. 5:**
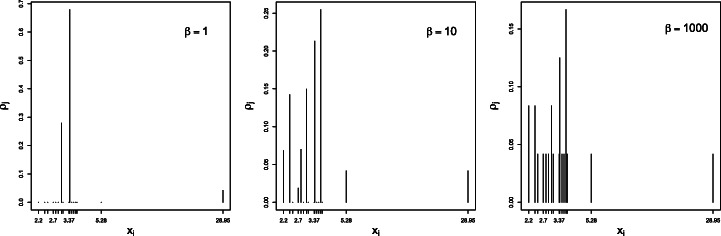
For decreasing temperature (that is for increasing *β*, from the left to the right panels), more percepts tend to emerge. Right: in the low-temperature limit, the histogram of the percepts coincides with the original copper concentration data, that is *ρ*_*j*_ = *f*_*j*_

The medium-temperature regime, neither characterized by the presence of all percepts nor the sole presence of dominating percepts, is arguably the most convoluted: by increasing the temperature, percepts can disappear, then reappear later (Figs. 1, 3 and 5). However, fairly simple and revealing relations still exist for the percept banality *τ*_*i*_(***f***) in (8), considered as a function of ***f*** (that is for ***S*** fixed and evaluated on the optimal transition ***Z***(***f***)):

#### **Theorem 5** (derivatives of the percept banalities)

For all *β* > 0, the derivatives of the percept banalities with respect to the item weights satisfy

26 $$  \frac{1}{\tau_{i}}\frac{\partial \tau_{i}}{\partial f_{j}}=\frac{1}{\tau_{j}}\frac{\partial \tau_{j}}{\partial f_{i}}=\sum\limits_{k} \frac{f_{k}}{{\tau_{k}^{2}}}\frac{\partial \tau_{k}}{\partial f_{i}}\frac{\partial \tau_{k}}{\partial f_{j}} \qquad\text{and}\qquad \sum\limits_{k} \frac{f_{k}}{\tau_{k}}\frac{\partial^{2} \tau_{k}}{\partial f_{i}\partial f_{j}}=0. $$

Identities (26) permit to prove the concavity of the effective entropy for all *β* > 0 (Theorem 9). Arguably relevant in further perturbation studies, they are not further interpreted nor investigated here.

## Types of Dissimilarities and Illustrations

### Example B: World Cities

Sparsely populated places, spatially close to more populated places, tend to be overlooked, and designated by the latter — a mechanism captured by the present formalism.

The population *N*_*i*_, latitudes *ϕ*_*i*_, longitudes *𝜃*_*i*_ of *n* = 30 world cities have been extracted from the (now outdated) R dataset world.cities{maps}. The selected sample contains the five most populated cities for each of the six continents. Relative weights are *f*_*i*_ = *N*_*i*_/*N*_∙_ and geodesic distances are given by

27 $$ \begin{array}{@{}rcl@{}} d_{ij}^{\mathtt{geo}} &=&\arccos(\kappa_{ij}) \\ \text{where}\qquad\kappa_{ij} &=&\sin\phi_{i}\sin\phi_{j}+\cos\phi_{i}\cos\phi_{j}\cos(\theta_{i}-\theta_{j}). \end{array} $$

**Fig. 6:**
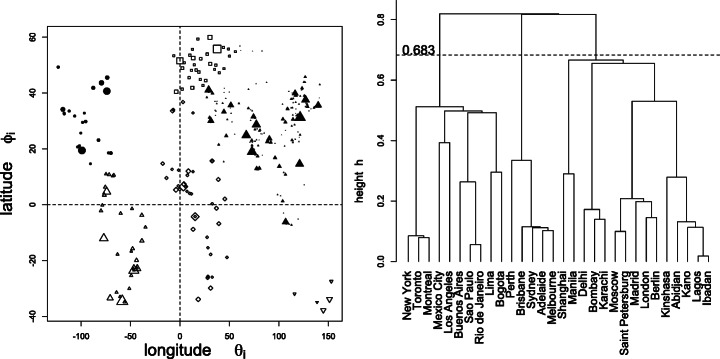
Left: cyclindrical projection of the 313 world cities with more than 10^6^ inhabitants, where the size of symbols reflects the population. The five most populated cities of each continent form the dataset of *n* = 30 objects, weighted by their population size. Right: dendrogram for the *n* = 30 retained cities, resulting from single linkage on ***D***, where ***D*** are the geodesic distances given in (27). The ultrametric dissimilarities $d^{\mathtt {ultra}_{ij}}$ were obtained as the height at which *i* and *j* are merged. Cutting the dendrogram as above (at 0.683) yields three groups (Americas, Oceania, Africa-Asia-Europe)

The left panel of Fig. 6 depicts the corresponding cylindrical projection, and the right panel depicts the dendrogram resulting from the *single linkage* *hierarchical ascendent classification* (HAC) applied on dissimilarities ***D***^geo^.

**Fig. 7:**
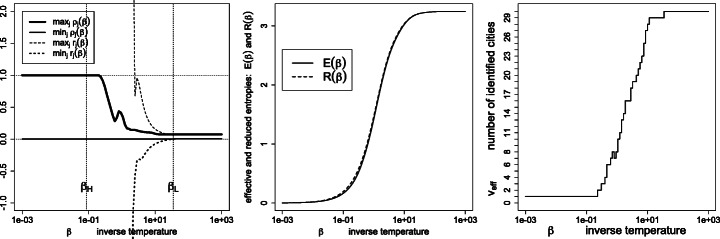
Similarities $\boldsymbol {S}=\exp (- \beta \boldsymbol {D}^{\texttt {geo}})$, where ***D***^geo^ are the geodesic distances (27): signed weights ***r*** and percept weights ***ρ*** for the various regimes (left panel), effective and reduced entropies (middle panel), and the effective variety (right panel)

**Fig. 8:**
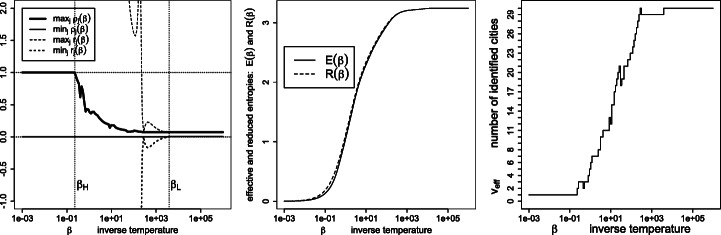
Same legend as Fig. 7, for similarities $\boldsymbol {S}=\exp (- \beta \boldsymbol {D}^{\texttt {geo2}})$, where $d_{ij}^{\texttt {geo2}}=(d_{ij}^{\texttt {geo}})^{2}$

**Fig. 9:**
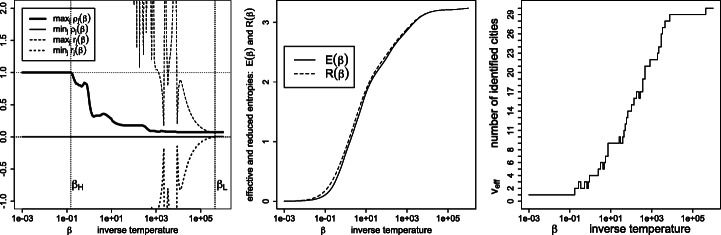
Same legend as Fig. 7, for similarities $\boldsymbol {S}=\exp (- \beta \boldsymbol {D}^{\texttt {geo3}})$, where $d_{ij}^{\texttt {geo3}}=(d_{ij}^{\texttt { geo}})^{3}$

**Fig. 10:**
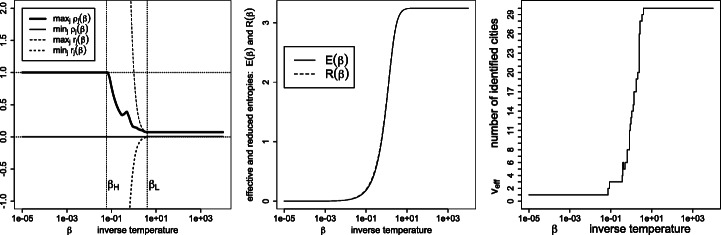
Same legend as Fig. 7, for similarities $\boldsymbol {S}=\exp (- \beta \sqrt {\boldsymbol {D}^{\texttt {geo}}})$

**Fig. 11:**
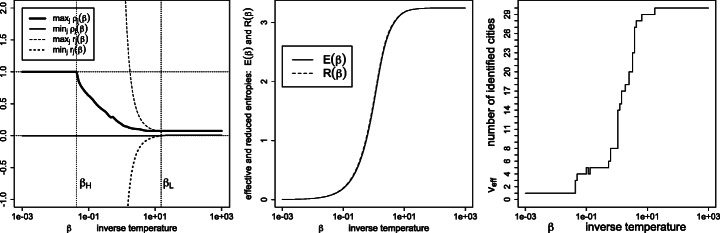
Same legend as Fig. 7, for similarities $\boldsymbol {S}=\exp (- \beta \boldsymbol {D}^{\texttt {ultra}})$, where ***D***^ultra^ is the ultrametric dissimilarity obtained from single linkage applied on ***D***^geo^ (Fig. 6 right panel)

**Fig. 12:**
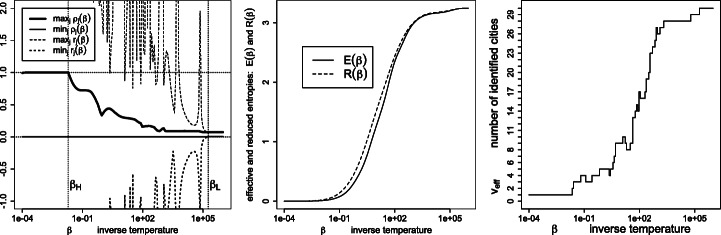
Same legend as Fig. 7, for similarities $\boldsymbol {S}=\exp (- \beta \boldsymbol {D}^{\texttt {random}})$, where the symmetric off-diagonal components of the random dissimilarity ***D***^random^ (see the text)

Figures 7, 8, 9, 10, 11 and 12 give, keeping the city weights ***f*** unchanged, the behavior of the signed weights ***r*** and percept weights ***ρ*** for the various regimes, as well as the effective and reduced entropies, and the effective variety. They do so, respectively, for the dissimilarities ***D***^geo^, their square, their cube, their squared root, ***D***^ultra^ (the ultrametric dissimilarity obtained from the dendrogram of Fig. 6) and for random dissimilarities ***D***^random^ (whose univariate coordinates are independently drawn as the square of a Student variate with three degrees of freedom).

The understanding of the class of *exponential similarity matrices* of the form $\boldsymbol {S}=\exp (-\beta \boldsymbol {D})$ (componentwise or Hadamard exponential), where *β* > 0 and ***D*** is a dissimilarity matrix, benefits from a wealth of acute studies in matrix analysis (see, e.g., Horn & Johnson, 1991; Critchley & Fichet, 1994; Martínez et al., 1994; Bapat & Raghavan, 1997; Deza & Laurent, 1997; Reams, 1999; Bavaud, 2009; Dellacherie et al., 2014 and references therein). Theorem 6 summarizes some salient features from these sources:

#### **Theorem 6** (exponential similarity matrices)

Let $\boldsymbol {S}=\exp (-\beta \boldsymbol {D})$. Then

- *6a)* ***S*** is positive semi-definite (p.s.d.) for all *β* > 0 iff ***D*** is squared Euclidean.
- *6b)* ***S*** is positive definite (p.d.) for all *β* > 0, and hence inversible, iff ***D*** is squared Euclidean and proper.
- *6c)* if ***D*** is ultrametric (and hence squared Euclidean) and proper, then ***S***^− 1^ = (*s*^*ij*^) is, for all *β* > 0, a strictly diagonally dominant Stieltjes matrix, that is a p.d. matrix with *s*^*ij*^ ≤ 0 for *i*≠*j* and *s*^*i*∙^ > 0.
- *6d)* proper ultrametric dissimilarities possess an inverse (***D***^ultra^)^− 1^ = (*d*^*ij*^) obeying *d*^*i*∙^ > 0.

Recall that ***D*** = (*d*_*ij*_) is squared Euclidean iff ***D*** is of the form *d*_*ij*_ = ∥***x***_*i*_ −***x***_*j*_∥^2^ for some vectors $\boldsymbol {x}_{i} \in \mathbb {R}^{p}$. The dissimilarities ***D***^geo^ and $\sqrt {\boldsymbol {D}^{\texttt {geo}}}$ (Hadamard square root) are squared Euclidean (see, e.g., Critchley & Fichet, 1994), in contrast to ***D***^geo2^, ***D***^geo3^ or ***D***^random^ which are not. The difference between the reduced and effective entropies appears smaller for squared Euclidean dissimilarities, and still smaller for ultrametric dissimilarities which are investigated next.

### Ultrametric (Dis)similarities

The single-linkage ultrametric dissimilarity ***D***^ultra^ extracted from the dendrogram in Fig. 6 is squared Euclidean too (see, e.g., Critchley & Fichet, 1994), and yields a *strictly ultrametric similarity* satisfying $s_{ij}\ge \max \limits (s_{ik},s_{jk})$, as well as *s*_*ii*_ > *s*_*ij*_ for all *i*≠*j*.

From Theorem (6d), the normalized *circumweights* defined by

28 $$ \begin{array}{@{}rcl@{}} g_{i} & =&\frac{d^{i\bullet}}{d^{\bullet\bullet}}>0 \\ \text{satisfy}\qquad {\partial_{j}^{g}} & =&\sum\limits_{i} g_{i} d_{ij}=\frac{1}{d^{\bullet\bullet}}\sum\limits_{i} d^{i\bullet}d_{ij}=\frac{1}{d^{\bullet\bullet}}=2{{\varDelta}}^{g}. \end{array} $$

In other terms, there exists, for each proper ultrametric dissimilarity, a weight distribution ***g*** such that the barycenter of the configuration is at *equal distanceΔ*^*g*^ = 1/(2*d*^∙∙^) from all items, which lie on the circumcircle centered on the barycenter. This vividly illustrates the poor low-dimensional compressibility of ultrametric configurations when performing multidimensional scaling. This distance turns out to be 0.683 in the world cities example (see the right panel of Fig. 6), corresponding to a geodesic distance of about 0.683 × 6371 ≅ 4351km.

**Fig. 13:**
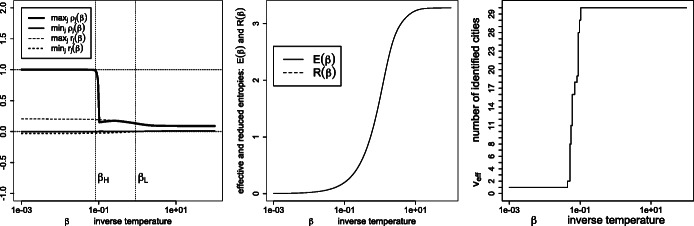
Same legend as Fig. 7, for similarities $\boldsymbol {S}=\exp (- \beta \boldsymbol {D}^{\texttt {ultra}})$ and item weights given by the circumweights ***g*** in (28)

When endowed with circumweights, all items dominate by construction in the high-temperature regime (i.e., *A*^⋆^ = 〈*n*〉), but they do not necessarily appear in the medium-temperature regime (Fig. 13). As pointed out by Pavoine et al. (2005), ultrametric dissimilarities grant that the maximum value of Rao quadratic entropy *Δ*(***f***) is precisely attained for ***f*** = ***g***, where all items or species are present (i.e., supp(***g***) = 〈*n*〉), as expected from a decent measure of biodiversity.

**Fig. 14:**
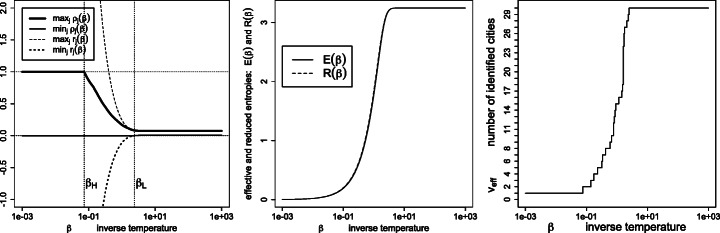
Same legend as Fig. 7, for equidistant dissimilarities *d*_*ij*_ = *d* > 0 for *i*≠*j*, and city weights ***f*** unchanged

Figure 14 depicts the case of ultrametric *equidistant similaritiesd*_*ij*_ = *d* > 0 for *i* ≠ *j*, for which the critical temperatures can be explicitly computed:

#### **Theorem 7** (equidistant dissimilarities)

Let *d*_*ij*_ = *d* > 0 for *i*≠*j* (and *d*_*ii*_ = 0), with distinct weights ***f*** decreasingly ordered as *f*_[1]_ > *f*_[2]_ > … > *f*_[*n*]_. Let $s(\beta )=\exp (-\beta d/{{\varDelta }})$. The low-temperature regime holds for *s*(*β*) ∈ [0,*s*_−_), and the high-temperature regime holds for *s*(*β*) ∈ (*s*_+_,1), where

$$ s_-=\frac{f_{[n]}}{1-(n-1)f_{[n]}} \qquad\quad < \quad \qquad s_+=\frac{f_{[2]}}{f_{[1]}}. $$

Equivalenty,

$$ \beta_L=\frac{1}{2}\left( 1-\sum\limits_i f_i^{2}\right)\ln \frac{1-(n-1)f_{[n]}}{f_{[n]}} \qquad > \qquad \beta_H=\frac{1}{2}\left( 1-\sum\limits_i f_i^{2}\right)\ln \frac{f_{[1]}}{f_{[2]}}. $$

## Further Properties as Measures of Diversity

The exponential of the entropy $\exp (H)$ can be interpreted as a measure of the effective number of alternatives, taking on its maximum value *n* for uniform distributions *f*_*i*_ = 1/*n*. Similarly, the reduced entropy *R* has been introduced as a similarity-dependent measure of ecological diversity, where $\exp (R)$ (denoted ^1^*D*^*S*^(***f***) by Leinster and Cobbold, 2012) can be interpreted as an effective, similarity-reduced number of alternatives. However, neither *R*(***f***) nor *E*(***f***) take on their maximum on the uniform distribution in general (see Theorem 10 below).

The larger the similarity between items, the smaller should be the corresponding diversity. This *monotonicity* property is satisfied for the reduced and effective entropies: if $\tilde {s}_{ij}\ge s_{ij}$ for all *i*,*j*, then $\tilde {R}\le R$ (from (??)) and $\tilde {E}\le E$ (from (10)).

Another property shared by the reduced and effective entropies is the so-called *modularity* (Leinster & Cobbold 2012), to be compared with the decomposition of Shannon entropy under *aggregation*: let *X* be a categorical variable, and let *G* be a coarser variable resulting from the aggregation of some categories of *X*, that is *H*(*G*|*X*) = 0. Then $H(X)=H(G)+H(X|G)=H(G)+{\sum }_{g} \pi _{g} H(X|g)$. Here we have:

### **Theorem 8** (modularity)

Let the *n* items be partitioned into *m* communities or groups *g* = 1,…,*m*, in such a way that the similarity between two items belonging to different groups is zero. Then

29 $$  R=H(G)+\sum\limits_{g} \pi_{g} R^{g}\qquad\quad\text{and} \qquad\quad E=H(G)+\sum\limits_{g} \pi_{g} E^{g}, $$

where $\pi _{g}={\sum }_{i\in g}f_{i} $ is the relative weight of group *g*, $H(G)=-{\sum }_{g} \pi _{g} \ln \pi _{g}$, and the quantities *R*^*g*^ and *E*^*g*^ denote the reduced and effective entropies within group *g* only.

What if the rather restrictive conditions of the previous theorem, namely strict partitioning of items into groups (that is *H*(*G*|*X*) = 0) and zero inter-group similarity (that is $\boldsymbol {S}=\bigoplus _{g} \boldsymbol {S}^{g}$) are lifted? The question directly points to the issue of *concavity*, a desirable property of diversity measures, which says that the diversity of the whole is not less that the average diversity of its parts: such is, notably, the case for the variance (the total variance is bounded below by the within-groups variance, the difference being the between-groups variance), and Shannon entropy (*H*(*X*) ≥ *H*(*X*|*G*)).

The effective entropy turns out to be concave as well (Theorem 9 below). Let ${f_{i}^{g}}$ (obeying ${f_{i}^{g}}\ge 0$ and $f_{\bullet }^{g}=1$) denote the relative weight of item *i* in group *g* = 1,…,*m*, let *π*_*g*_ ≥ 0 with *π*_∙_ = 1 denote the relative importance of group *g*, and let $f_{i}={\sum }_{g} \pi _{g} {f_{i}^{g}}$ denote the item weights of the whole. Also, let *F*[***f***,***Z***] denote the free energy (10) considered as a function of the two independent variables ***f*** and ***Z***, and finally let $E(\boldsymbol {f})=\min \limits _{\boldsymbol {Z}\in {\mathcal Z}}F[\boldsymbol {f},\boldsymbol {Z}]$.

### **Theorem 9** (concavity of the effective entropy)

The effective entropy is *concave* in ***f***, that is

30 $$  E(\boldsymbol{f})\ge \sum\limits_{g=1}^{m} \pi_{g}  E(\boldsymbol{f}^{g})\qquad\text{for}\quad \boldsymbol{f}=\sum\limits_{g} \pi_{g} \boldsymbol{f}^{g}. $$

As the proof in the Appendix makes clear, virtually no condition on ***S*** in (10) is required to ensure the validity of Theorem 9. By contrast, the concavity of the reduced entropy *R*(***f***), although supposedly simpler to manipulate than *E*(***f***), seems harder to establish: without additional conditions on ***S***, *R*(***f***) is *not concave in general*, as shown by the following counter-example: let *n* = 3 and

$$ \boldsymbol{S}=\left( \begin{array}{lll}1 & 0 & 0.7 \\0 & 1 & 0.7 \\ 0.7 & 0.7 & 1 \end{array}\right) \boldsymbol{f}=\left( \begin{array}{c}0.25 \\0.25 \\0.5 \end{array}\right) \boldsymbol{f}^1=\left( \begin{array}{c}0.2 \\0.2 \\0.6 \end{array}\right) \boldsymbol{f}^{2}=\left( \begin{array}{c}0.3 \\0.3 \\0.4 \end{array}\right). $$

Then $\boldsymbol {f}=\frac {1}{2}(\boldsymbol {f}^{1}+\boldsymbol {f}^{2})$ and $R(\boldsymbol {f})-\frac {1}{2} R(\boldsymbol {f}^{1})-\frac {1}{2} R(\boldsymbol {f}^{2})=0.33667-\frac {1}{2}\cdot 0.26791-\frac {1}{2}\cdot 0.40622=-0.00039$, whose negative sign violates the concavity of *R*(***f***). By contrast, one finds $E(\boldsymbol {f})-\frac {1}{2} E(\boldsymbol {f}^{1})-\frac {1}{2} E(\boldsymbol {f}^{2})=0.214-\frac {1}{2}\cdot 0.143-\frac {1}{2}\cdot 0.214=0.036$, non-negative as it must.

In the naive approach, the uniform distribution *f*_*i*_ = 1/*n* is well-known to maximize Shannon entropy *H*(***f***). This is not the case anymore for the effective entropy, whose non-uniform maximizing distribution ***f***^∘^ can be explicitly computed for low temperatures:

### **Theorem 10** (maximum effective entropy)

Let *β* be large enough so that ***S*** is invertible with *s*^*i*∙^ > 0 for all *i*. Then the item distribution $f^{\circ }_{i}=s^{i\bullet }/s^{\bullet \bullet }$ maximizes the effective entropy $E(\boldsymbol {f})\le E(\boldsymbol {f}^{\circ })=\ln s^{\bullet \bullet }$. Also, ***f***^∘^ constitutes a stationary point of the reduced entropy *R*(***f***). Furthermore, *E*(***f***^∘^) = *R*(***f***^∘^), $\tau _{i}^{\circ }=1/s^{\bullet \bullet }$, $\rho _{j}^{\circ }=f^{\circ }_{j}$ and $z_{ij}^{\circ }=s_{ij}s^{j\bullet }$.

Thus $\exp (E(\boldsymbol {f}^{\circ }))=s^{\bullet \bullet }$ measures, for *β* large, the maximum number of effective items, and tends to *n*, as it must, in the naive limit $\beta \to \infty $. By contrast, ***f***^∘^ is a stationary point of the reduced entropy *R*(***f***), whose lack of concavity cannot however exclude the presence of multiple extrema in general.

Finally, we address the question of the *subadditivity* of the effective and reduced entropies, which expresses for Shannon entropy as the independence bound *H*(*X*,*Y* ) ≤ *H*(*X*) + *H*(*Y* ), where equality holds iff *X* and *Y* are independent. Here *X* can be thought of as a categorical variable whose modalities *i* = 1,…,*n* are similar in part, as expressed by the *n* × *n* similarity matrix $s_{ij}^{X}$. Likewise, *Y* is a categorical variable with modalities *k* = 1,…,*m* and associated *m* × *m* similarity matrix $s_{kl}^{Y}$. The weights of the cross-modalities are $f_{(ik)}^{XY}$ with ${f_{i}^{X}}=f_{(i\bullet )}^{XY}$ and ${f_{k}^{Y}}=f_{(\bullet k)}^{XY}$, and we *assume* the similarities between cross-modalities to be defined as

31 $$  s_{(ik)(jl)}^{XY}=s_{ij}^{X}\cdot s_{kl}^{Y}\qquad\text{that is}\quad \boldsymbol{S}^{XY}=\boldsymbol{S}^{X}\otimes \boldsymbol{S}^{Y}\quad\text{(Kronecker product)} $$

which is equivalent to the definition $d_{(ik)(jl)}^{XY}=d_{ij}^{X}+d_{kl}^{Y}$.

### **Theorem 11** (subadditivity)

Consider a bivariate distribution ***f***^*XY*^ with margins ***f***^*X*^ and ***f***^*Y*^, and let $\hat {f}^{XY}_{(ik)}={f^{X}_{i}} {f^{Y}_{k}}$ be the corresponding independent distribution. Then

$$ E\left( \hat{\boldsymbol{f}}^{XY}\right)=E\left( \boldsymbol{f}^{X}\right)+E\left( \boldsymbol{f}^{Y}\right)\qquad\quad{\text{and}}\qquad\quad R\left( \hat{\boldsymbol{f}}^{XY}\right)=R\left( \boldsymbol{f}^{X}\right)+R\left( \boldsymbol{f}^{Y}\right). $$

Furthermore, $E\left (\boldsymbol {f}^{XY}\right )\le E\left (\hat {\boldsymbol {f}}^{XY}\right )$, which makes *E* subadditive.

As the proof in the Appendix makes clear, subadditivity does not hold for the reduced entropy, which, after its lack of concavity (Theorem 9) suffers here a second setback. For a minimal example, consider *n* = *m* = 2 and

$$ \boldsymbol{S}^X=\left( \begin{array}{cc}1 & .8 \\.8 & 1 \end{array}\right) \boldsymbol{S}^Y=\left( \begin{array}{cc}1 & .5 \\.5 & 1 \end{array}\right) \left( f_{(ik)}^{XY}\right)=\left( \begin{array}{cc}.41& .29 \\.19 & 11 \end{array}\right) \left( \hat{f}_{(ik)}^{XY}\right)=\left( \begin{array}{cc}.42& .28 \\.18 & .12 \end{array}\right). $$

with ***f***^*X*^ = (.7,.3) and ***f***^*Y*^ = (.6,.4). Then $E\left (\boldsymbol {f}^{XY}\right )-E\left (\boldsymbol {f}^{X}\right )-E\left (\boldsymbol {f}^{Y}\right )=-1.7\cdot 10^{-16}<0$, but $R\left (\boldsymbol {f}^{XY}\right )-R\left (\boldsymbol {f}^{X}\right )-R\left (\boldsymbol {f}^{Y}\right )=1.2\cdot 10^{-5}>0$.

## Conclusion

This paper has introduced and analyzed the properties of a new diversity index, the effective entropy. It is based upon a formalism which is both tractable and non-trivial, and it is no coincidence that parts of this formalism also serve in statistical mechanics, statistics, operation research and information theory for the exposition and solution of their own relevant issues. The effective entropy appears to satisfy many properties expected from a diversity index, and handles the presence of item (dis)similarites in a systematic, controlled way. It remedies the formal deficiencies of the reduced entropy, for which it provides a lower bound, and provides an explicit mechanism of diversity reduction due to the confusion between close items.

For a configuration of weighted items with given pair dissimilarities, the effective and the reduced entropies constitute a one-parameter diversity family indexed by a discriminability or inverse temperature parameter. Both entropies converge towards Shannon entropy in the low-temperature limit, provided items are pairwise distinct. In the high-temperature limit, the behavior of the effective and reduced entropy is governed by the minimum eccentricity, respectively the average eccentricity, that is (up to a factor two) Rao quadratic entropy.

Identifying the exact conditions on the (dis)similarities making the reduced entropy concave or subadditive constitutes an obvious future challenge. Determining a well-behaved “power” generalization of the presently proposed “logarithmic” effective dissimilarity, in the spirit of Rényi or Tsallis entropies, constitutes another.

## Appendix

### *Proof of *$\lim _{\beta \to \infty }E=H_{\text {\scriptsize agg}}$*for semi-proper dissimilarities*

(15): for $\beta \to \infty $, the energy term (3) must vanish, making *z*_*i**j*_ = 0 whenever *i* and *j* belong to different equivalence classes *C*[*i*]≠*C*[*j*]. For *i*,*j* in the same class *C*, the mutual information (4) is minimum under independence (within *C*), that is for *z*_*i**j*_ = *a*_*j*_; identity $\rho _{j}={\sum }_{i} f_{i} z_{ij}={\sum }_{i\in C}f_{i} a_{j}=F_{C} a_{j}$ entails

$$ K=\sum\limits_{C} \sum\limits_{i,j\in C}f_{i} \frac{\rho_{j}}{F_{C}}\ln \frac{\rho_{j}}{F_{C}\rho_{j}}=-\sum\limits_{C} \sum\limits_{i\in C}f_{i} \underbrace{\sum\limits_{j\in C}\frac{\rho_{j}}{F_{C}}}_{=1}\ln F_{C}= -{\sum}_{C}F_{C}\ln F_{C}=H_{\text{\scriptsize agg}}. $$

□

### *Proof of Theorem 1*

Differentiating $F[\boldsymbol {Z}]+{\sum }_{i} \lambda _{i} (1-z_{i\bullet })$ with respect to *z*_*i**j*_ (where *λ*_*i*_ is the Lagrange multiplier associated with constraint *z*_*i*∙_ = 1) yields the first-order condition $\beta d_{ij}+\ln \frac {z_{ij}}{\rho _{j}}=\lambda _{i}/f_{i}$, equivalent to (8); note that non-negativity of ***Z*** is automatically ensured. The second derivative $\frac {\partial ^{2}F[\boldsymbol {Z}]}{\partial z_{ij}\partial z_{i^{\prime }j^{\prime }}}=\delta _{jj^{\prime }}(\frac {f_{i}\delta _{ii^{\prime }}}{z_{ij}}-\frac {f_{i}f_{i^{\prime }}}{\rho _{j}})$ (see, e.g., Bavaud, 2009) can be shown to be positive semi-definite. Eigenvalues are zero for eigenvectors parallel to *z*_*i**j*_ (that is ${\sum }_{i^{\prime }}\frac {\partial ^{2}F[Z]}{\partial z_{ij}\partial z_{i^{\prime }j^{\prime }}}z_{i^{\prime }j}=0)$, but positive otherwise, implying the strict convexity of *F*[***Z***] under admissible variations *z*_*i**j*_ →*z*_*i**j*_ + *ε*_*i**j*_ with *ε*_*i*∙_ = 1, and thus the unicity of the optimal solution in the interior of the convex set ${\mathcal Z}$. Summing the first identity in (7) over ${\sum }_{i} f_{i}\ldots $ yields *ρ*_*j*_ = *c*_*j*_*ρ*_*j*_, that is *c*_*j*_ = 1 for *ρ*_*j*_ > 0.

The above derivation is incomplete at the boundary of ${\mathcal Z}$ when *ρ*_*j*_ = 0, for some *j* (implying *z*_*i**j*_ = 0 for all *i*). Here the condition *c*_*j*_ ≤ 1 can be derived (with some additional work) by starting from the Kuhn-Tucker conditions of convex optimization, or directly as follows: adding mass on percept *j* can be performed by the transformation $\tilde {z}_{ij}=\alpha _{i}\in (0,1)$ and $\tilde {z}_{ik}=(1-\alpha _{i})z_{ik}$ for *k*≠*j*. The new free energy reads $F[\tilde {\boldsymbol {Z}}]=F[\boldsymbol {Z}]+\bar {\alpha }\zeta (\gamma )+0(\alpha ^{2})$ where $\bar {\alpha }={\sum }_{i} f_{i} \alpha _{i}$, $\gamma _{i}=f_{i} \alpha _{i}/\bar {\alpha }\ge 0$ and $\zeta (\gamma )={\sum }_{i} \gamma _{i} [\beta d_{ij}+\ln \tau _{i} +\ln (\gamma _{i}/f_{i})]$ is convex in *γ*. Hence *ρ*_*j*_ = 0 minimizes the free energy if $\min \limits _{\gamma }\zeta (\gamma )=\zeta (\gamma ^{0})\ge 0$. One finds ${\gamma ^{0}_{i}}=f_{i}s_{ij}/(\tau _{i} c_{j})$ and $\zeta (\gamma ^{0})=-\ln c_{j}$, and the latter condition becomes *c*_*j*_ ≤ 1. □

### *Proof of Theorem 2*

Consider the functional $M[\boldsymbol {Z},\boldsymbol {\rho }]={\sum }_{ij}f_{i}z_{ij}(\beta d_{ij}+\ln \frac {z_{ig}}{\rho _{j}})$, where ***Z*** is a transition matrix and ***ρ*** some distribution. Then (a) $\boldsymbol {\rho }^{(t)}=\arg \min \limits _{\boldsymbol {\rho }} M[\boldsymbol {Z}^{(t)},\boldsymbol {\rho }]$ and (b) $\boldsymbol {Z}^{(t+1)}=\arg \min \limits _{\boldsymbol {Z}} M[\boldsymbol {Z},\boldsymbol {\rho }^{(t)}]$. Hence

$$ F\left[\boldsymbol{Z}^{(t+1)}\right]=M\left[\boldsymbol{Z}^{(t+1)},\boldsymbol{\rho}^{(t+1)}\right]\stackrel{(a)}{\le} M\left[\boldsymbol{Z}^{(t+1)},\boldsymbol{\rho}^{(t)}\right]\stackrel{(b)}{\le} M\left[\boldsymbol{Z}^{(t)},\boldsymbol{\rho}^{(t)}\right]=F\left[\boldsymbol{Z}^{(t)}\right] $$

Applying ${\sum }_{i} f_{i} {\cdots } $ to the second identity in (19) yields $\lim _{t\to \infty } \rho ^{(t+1)}_{j}/\rho ^{(t)}_{j}=~c_{j}$. Thus *c*_*j*_ < 1 implies $\rho _{j}=\lim _{t\to \infty }\rho ^{(t)}_{j}=0$, in accordance with Theorem (1).

The condition $\boldsymbol {Z}^{(0)}\in {\mathcal Z}_{\langle n \rangle }$ is crucial, since restricting ***Z***^(0)^ to ${\mathcal Z}_{A}$, say, where *A* is a strict subset of 〈*n*〉, that is starting with a ***Z***^(0)^ with column margins $z^{(0)}_{\bullet j}=0$ for *j*∉*A*, will automatically generate subsequent transitions $\boldsymbol {Z}^{(t)}\in {\mathcal Z}_{A}$, converging to the optimal solution of the *restricted problem*

$$ E_A=\min_{\boldsymbol{Z}\in {\mathcal Z}_A}F[\boldsymbol{Z}]\ge E=\min_{\boldsymbol{Z}\in {\mathcal Z}}F[\boldsymbol{Z}], $$

corresponding to a so-called *metastable solution* in statistical mechanics, here betrayed by the possible presence of some *c*_*j*_ > 1 for *j*∉*A*, in violation of Theorem 1.

The iterative process (19) has been rediscovered several times in various disciplines. Known as the Blahut-Arimoto algorithm in information theory, it constitutes a variant of the EM algorithm of Statistics, which (among other purposes) serves at identifying the optimal soft clustering minimizing the functional $F^{\text \tiny \texttt {cluster}}=\beta {\sum }_{i=1}^{n}{\sum }_{g=1}^{m} f_{i} z_{ig} d_{ig}+K[\boldsymbol {Z}]$. Here *z*_*i**g*_ ≥ 0 (with *z*_*i*∙_ = 1) is the membership probability of item *i* in *cluster* *g* = 1,…,*m*, and *d*_*i**g*_ is the dissimilarity between *i* and the optimal cluster representative, which turns out to be its weighted barycenter for squared Euclidean dissimilarities (see, e.g., Bavaud, 2009) and references therein). In contrast to problem (6), the cluster representatives *g* are not constrained to *g* ∈〈*n*〉: for *m* ≥*n*, the minimum free energy *F*^cluster^ provides a *lower bound* for the effective entropy (6). □

### *Proof of Theorem 3*

By Theorem 1, *c*_*j*_ = 1 whenever *ρ*_*j*_ > 0, thus ***S******γ*** = ***1*** where *γ*_*i*_ = *f*_*i*_/*τ*_*i*_ by (17). Multiplying by ***S***^− 1^ yields *f*_*i*_/*τ*_*i*_ = *s*^*i*∙^, and hence ***ρ*** = ***S***^− 1^***τ***, that is $\rho _{j}={\sum }_{i} f_{i} s^{ij}/s^{i\bullet }$ which is (21). The third and fourth identities of (22) are obtained by simple substitution from (7), resp. (14). Identities ${\sum }_{j} z_{ij}=1$ and ${\sum }_{i} f_{i} z_{ij}=\rho _{j}$ are straightforward to demonstrate, showing (21) and (22) to yield the optimal solution, provided $\min \limits _{j}r_{j}\ge 0$. □

### *Proof of Theorem 4*

(24) and (25) follow from the properties discussed in the beginning of Section 3.4. For *β* = 0, ***S*** = ***J*** and all sub-indicators (17) are identically one. First-order expansion *s*_*i**j*_ = 1 −*β**d*_*i**j*_ + 0(*β*^2^) yields

$$ c_j(\beta)=\sum\limits_i f_i (1-\beta d_{ij}+\beta\sum\limits_{k\in A^{\star}}\rho_k d_{ik})+0(\beta^{2})=1-\beta(\partial_j-\partial^{\star})+0(\beta^{2}) $$

which demonstrates the stability of the solution. The high-temperature expansions (25), as well as the exactness of the relation *E* = *β**∂*^⋆^ when *A*^⋆^ consists of a single element, follows analogously from (10). □

### *Proof of Theorem 5*

Following Theorem 1, *c*_*j*_ = 1 for *ρ*_*j*_ > 0. Derivating (17) with respect to *f*_*k*_, and a second time with respect to *f*_*l*_, yields *for* *ρ*_*j*_ > 0

A.1 $$ \begin{array}{@{}rcl@{}} \frac{s_{kj}}{\tau_{k}}& = &\sum\limits_{i} \frac{f_{i}s_{ij}}{{\tau_{i}^{2}}}\frac{\partial \tau_{i}}{\partial f_{k}} \end{array} $$

A.2 $$ \begin{array}{@{}rcl@{}} \frac{s_{kj}}{{\tau_{k}^{2}}}\frac{\partial \tau_{k}}{\partial f_{l}}+ \frac{s_{lj}}{{\tau_{l}^{2}}}\frac{\partial \tau_{l}}{\partial f_{k}}& = & 2\sum\limits_{i} \frac{f_{i}s_{ij}}{{\tau_{i}^{3}}}\frac{\partial \tau_{i}}{\partial f_{k}}\frac{\partial \tau_{i}}{\partial f_{l}}- \sum\limits_{i} \frac{f_{i}s_{ij}}{{\tau_{i}^{2}}}\frac{\partial^{2} \tau_{i}}{\partial f_{k}\partial f_{l}} \end{array} $$

Multipying both identities with *ρ*_*j*_ (either positive or zero) and summing over *j* yields using (8) the following identities valid for all *k*,*l*

A.3 $$ \begin{array}{@{}rcl@{}} 1& = &\sum\limits_{i} \frac{f_{i}}{\tau_{i}}\frac{\partial \tau_{i}}{\partial f_{k}} \end{array} $$

A.4 $$ \begin{array}{@{}rcl@{}} \frac{1}{\tau_{k}}\frac{\partial \tau_{k}}{\partial f_{l}}+ \frac{1}{\tau_{l}}\frac{\partial \tau_{l}}{\partial f_{k}}& = & 2\sum\limits_{i} \frac{f_{i}}{{\tau_{i}^{2}}}\frac{\partial \tau_{i}}{\partial f_{k}}\frac{\partial \tau_{i}}{\partial f_{l}}- \sum\limits_{i} \frac{f_{i}}{\tau_{i}}\frac{\partial^{2} \tau_{i}}{\partial f_{k}\partial f_{l}}. \end{array} $$

On the other hand, derivating (A.3) with respect to *f*_*l*_ yields

A.5 $$  \frac{1}{\tau_{l}}\frac{\partial \tau_{l}}{\partial f_{k}}= \sum\limits_{i} \frac{f_{i}}{{\tau_{i}^{2}}}\frac{\partial \tau_{i}}{\partial f_{k}}\frac{\partial \tau_{i}}{\partial f_{l}}- \sum\limits_{i} \frac{f_{i}}{\tau_{i}}\frac{\partial^{2} \tau_{i}}{\partial f_{k}\partial f_{l}}. $$

The symmetry in $k\leftrightarrow l$ in the r.h.s. of (A.5) implies $\frac {1}{\tau _{l}}\frac {\partial \tau _{l}}{\partial f_{k}}=\frac {1}{\tau _{k}}\frac {\partial \tau _{k}}{\partial f_{l}}$. Substituting the latter in (A.4) and comparing with (A.5) finally yields ${\sum }_{i} \frac {f_{i}}{\tau _{i}}\frac {\partial ^{2} \tau _{i}}{\partial f_{k}\partial f_{l}}=0$. □

### *Proof of Theorem 6*

A proof of (6a) can be found in Horn & Johnson (1991 p. 476), Bapat & Raghavan (1997 p. 163) or in Reams (1999), and applies to conditionally semi-negative definite (c.s.n.d.) matrices, that is to symmetric *n* ×*n* matrices ***D*** = (*d*_*i**j*_) such that ${\sum }_{ij}d_{ij}h_{i}h_{j}\le 0$ for all *h* with *h*_∙_ = 0. On the other hand, ***D*** is squared Euclidean iff it is c.s.n.d. with a null diagonal (see, e.g., Critchley and Fichet, 1994; Deza & Laurent, 1997). Lemma 2.5 in Reams (1999) demonstrates (6b). Property (6c) and its proof appear in (Martínez et al., 1994); see also (Dellacherie et al., 2014) and references therein. Property (6d) follows from adapting Lemma 1 in Martínez et al. (1994) to ***D***^ultra^ instead of ***S*** (see also Pavoine et al., 2005 and references therein). □

### *Proof of Theorem 7*

After decreasing labelling, *∂*_*j*_ = *d*(1 −*f*_*j*_), hence *j* = 1 dominates. Also, ${{\varDelta }}/d=\frac {1}{2}(1-{\sum }_{i} {f_{i}^{2}})$ and *τ*_*j*_ = *s* + (1 −*s*)*ρ*_*j*_ where *s* = *s*(*β*). Sub-indicators (17) are of the form *c*_*j*_ = *φ*(*s*) + (1 −*s*)*f*_*j*_/[*s* + (1 −*s*)*ρ*_*j*_], where the function *φ*(*s*) does not depend on *j*. Hence, for *j*≠ 1, *c*_*j*_ < *c*_1_ = 1 (and thus *ρ*_1_ = 1 and *ρ*_*j*_ = 0) iff *f*_*j*_/*s* < *f*_1_, that is iff $s>\max \limits _{j\neq 1}f_{j}/f_{1}=s_{+}$.

On the other hand, the inverse of ***S*** = (1 −*s*)***I***_*n*_ + *s****J***_*n*_ is $\boldsymbol {S}^{-1}=\alpha \boldsymbol {I}_{n}+\gamma \boldsymbol {J}_{n}$ where *α* = 1/(1 −*s*) and *γ* = −*s*/[(1 −*s*)(1 −*s* + *n**s*)]. The signed weights (21) read *r*_*j*_ = (*α**f*_*j*_ + *γ*)/(*α* + *n**γ*) and $\min \limits _{j} r_{j}>0$ iff *α**f*_*n*_ + *γ* > 0, that is iff *s* < *f*_*n*_/[1 − (*n* − 1)*f*_*n*_] = *s*_−_. □

### *Proof of Theorem 8*

The normalized item weights in group *g* are ${f_{i}^{g}}=f_{i}/\pi _{g}$ if *i* ∈*g*, and ${f_{i}^{g}}=0$ otherwise. By hypothesis, $\boldsymbol {S}=\bigoplus _{g} \boldsymbol {S}^{g}$, and thus, for *i* ∈*g*, $b_{i}={\sum }_{j\in g} f_{j} s_{ij}=\pi _{g}{\sum }_{j\in g} {f_{j}^{g}} s_{ij}^{g}=\pi _{g} {b_{i}^{g}}$. Then $R=-{\sum }_{i} f_{i} \ln b_{i}=-{\sum }_{g} \pi _{g} {\sum }_{i\in g}{f_{i}^{g}} \ln (\pi _{g} {b_{i}^{g}})=-{\sum }_{g} \pi _{g} \ln \pi _{g}-{\sum }_{g} \pi _{g} {\sum }_{i\in g}{f_{i}^{g}} \ln {b_{i}^{g}}=H(G)+{\sum }_{g} \pi _{g} R^{g}$.

Similarly, the normalized percept weights in group *g* are ${\rho _{j}^{g}}=\rho _{j}/\pi _{g}$ if *i* ∈*g*, and ${f_{i}^{g}}=0$ otherwise. For *i* ∈*g*, $\tau _{i}={\sum }_{j} s_{ij}\rho _{j} = \pi _{g} {\sum }_{j\in g} s^{g}_{ij}{\rho _{j}^{g}}= \pi _{g} {\tau _{i}^{g}}$. By hypothesis, the transitions *z*_*i**j*_ = *s*_*i**j*_*ρ*_*j*_/*τ*_*i*_ are zero if *i* and *j* belong to different groups; if they belong to the same group *g*, then $z_{ij}=z^{g}_{ij}=s^{g}_{ij}{\rho ^{g}_{j}}/{\tau ^{g}_{i}}$, where $z^{g}_{i\bullet }=1$ and ${\sum }_{i} {f_{i}^{g}} z_{ij}^{g}=\frac {1}{\pi _{g}}{\sum }_{i} f_{i} z_{ij} =\frac {1}{\pi _{g}}\rho _{j}= {\rho _{j}^{g}}$. Substituting the expression for $z_{ij}=z^{g}_{ij}$ in (10) yields

$$ \begin{array}{@{}rcl@{}} E& = & \sum\limits_{g} \sum\limits_{ij\in g}\pi_{g} {f_{i}^{g}}z_{ij}^{g}\ln \frac{z_{ij}^{g}}{s_{ij}^{g}\pi_{g} {\rho_{j}^{g}}}=-\sum\limits_{g} \pi_{g} \ln \pi_{g}+\sum\limits_{g} \pi_{g} \sum\limits_{ij\in g}{f_{i}^{g}}z_{ij}^{g} \ln \frac{z_{ij}^{g}}{s_{ij}^{g} {\rho_{j}^{g}}} =\\ & = & H(G)+\sum\limits_{g} \pi_{g} E^{g}. \end{array} $$

□

### *Proof Two Proofs of Theorem 9*

Taking into account *∂**ρ*_*j*_/*∂**f*_*k*_ = *z*_*k**j*_, the Hessian reads $\partial ^{2} F[\boldsymbol {f},\boldsymbol {Z}]/\partial f_{k}\partial f_{l}=-{\sum }_{j} z_{kj}z_{lj}/\rho _{j}$, which is negative semi-definite, thus establishing the concavity of *F*[***f***,***Z***] in ***f***. Thus $F[\boldsymbol {f},\boldsymbol {Z}]\ge {\sum }_{g} \pi _{g} F[\boldsymbol {f}^{g},\boldsymbol {Z}]$ and

$$ E(\boldsymbol{f})=\min_{\boldsymbol{Z}\in {\mathcal Z}}F[\boldsymbol{f},\boldsymbol{Z}]\ge\min_{\boldsymbol{Z}\in {\mathcal Z}}\left( \sum\limits_g \pi_g F[\boldsymbol{f}^g,\boldsymbol{Z}]\right)\ge\sum\limits_g \pi_g \min_{\boldsymbol{Z}\in {\mathcal Z}}F[\boldsymbol{f}^g,\boldsymbol{Z}]=\sum\limits_g \pi_g E(\boldsymbol{f}^g) $$

A second proof of Theorem 9 follows from Theorem 5: to establish the concavity of a quantity such as *E*(***f***), it is enough to consider two groups of equal importance $\pi _{1}=\pi _{2}=\frac {1}{2}$, such that ${f_{i}^{1}}=f_{i}+\eta _{i}$ and ${f_{i}^{2}}=f-\eta _{i}$, where ***η*** must satisfy ***η***_∙_ = 0 but can otherwise be chosen arbitrarily (provided it is small enough to insure $ {f_{i}^{1}},{f_{i}^{2}}\ge 0$; recall *f*_*i*_ > 0). The *excess*$\zeta (\boldsymbol {\eta }):=E(\boldsymbol {f})-\frac {1}{2}E(\boldsymbol {f}+\boldsymbol {\eta })-\frac {1}{2}E(\boldsymbol {f}-\boldsymbol {\eta })$ satisfies *ζ*(0) = 0 and

$$ \frac{\partial\zeta{\scriptstyle(\eta=0)}}{\partial \eta_i}=-\frac{1}{2}\frac{\partial E(\boldsymbol{f})}{\partial f_i}+\frac{1}{2}\frac{\partial E(\boldsymbol{f})}{\partial f_i}=0 \qquad\text{and}\qquad \frac{\partial^{2}\zeta{\scriptstyle(\eta=0)}}{\partial \eta_i\partial \eta_j}=-\frac{\partial^{2} E(\boldsymbol{f})}{\partial f_i\partial f_j}. $$

Thus, $\zeta (\boldsymbol {\eta })=-\frac {1}{2}{\sum }_{ij}E_{ij}(\boldsymbol {f})\eta _{i} \eta _{j} +0(\eta ^{3})$, and *ζ*(***η***) ≥ 0 in the neighborhood of ***η*** = 0 iff the Hessian *E*_*i**j*_(***f***) of *E*(***f***) is *conditionally negative semi-definite*, that is obeys ${\sum }_{ij}E_{ij}(\boldsymbol {f})\eta _{j} \eta _{j}\le 0$ for all ***η*** such that *η*_∙_ = 0.

Now (14), (A.3) and (26) yield

A.6 $$ \begin{array}{@{}rcl@{}} E_{i}(\boldsymbol{f}):=\frac{\partial E(\boldsymbol{f})}{\partial f_{i}}&=& -\ln \tau_{i}-\sum\limits_{k} \frac{f_{k}}{\tau_{k}} \frac{\partial\tau_{k}}{\partial f_{i}}=-\ln \tau_{i}-1 \end{array} $$

A.7 $$ \begin{array}{@{}rcl@{}} E_{ij}(\boldsymbol{f}):=\frac{\partial^{2} E(\boldsymbol{f})}{\partial f_{i}\partial f_{j}}& = & -\frac{1}{\tau_{i}}\frac{\partial\tau_{i}}{\partial f_{j}}=-\sum\limits_{k} \frac{f_{k}}{{\tau_{k}^{2}}}\frac{\partial \tau_{k}}{\partial f_{i}}\frac{\partial \tau_{k}}{\partial f_{j}} \end{array} $$

the later expression being obviously negative semi-definite, and hence conditionally negative semi-definite. □

### *Proof of Theorem 10*

For ***f*** = ***f***^∘^, one verifies in (21) that $r_{j}=f_{j}^{\circ }>0$, hence ***ρ***^∘^ = ***f***^∘^ by (22). As a result, (8) or (22) show $\tau _{i}^{\circ }=1/s^{\bullet \bullet }$ and finally $z_{ij}^{\circ }=s_{ij}s^{j\bullet }$. Since ***τ***^∘^ is constant, (A.6) together with $f_{i}^{\circ }>0$ then demonstrates ***f***^∘^ to be a stationary point for *E*(***f***), and hence, given the concavity of the latter, its unique maximizer, with value $E(\boldsymbol {f}^{\circ })=\ln s^{\bullet \bullet }$. Finally, identity $b^{\circ }_{i}=1/s^{\bullet \bullet }$ entails *R*(***f***^∘^) = *E*(***f***^∘^), and distribution *f*^∘^ is easily shown to constitue a stationary point of *R*(***f***). □

### *Proof of Theorem 11*

Let $\hat {b}^{XY}_{(ik)}$ denote the bivariate banalities evaluated at $\boldsymbol {f}^{XY}=\hat {\boldsymbol {f}}^{XY}$, with similar notations for the other quantities. Definition (31) immediately entails $\hat {b}^{XY}_{(ik)}={b_{i}^{X}}{b_{k}^{Y}}$ and thus $R\left (\hat {\boldsymbol {f}}^{XY}\right )=R\left (\boldsymbol {f}^{X}\right )+R\left (\boldsymbol {f}^{Y}\right )$. Turning to the effective entropy, one readily verifies that, whenever $\boldsymbol {f}^{XY}=\hat {\boldsymbol {f}}^{XY}$, the optimal joint transition $\hat {\boldsymbol {Z}}^{XY}$ obeying (7) is simply $\hat {\boldsymbol {Z}}^{XY}=\boldsymbol {Z}^{X}\otimes \boldsymbol {Z}^{Y}$, where ***Z***^*X*^ and ***Z***^*Y*^ are the corresponding univariate optimal transitions. In particular, $\hat {\rho }^{XY}_{(jl)}={\rho _{j}^{X}}{\rho _{l}^{Y}}$, $\hat {\tau }^{XY}_{(ik)}={\tau _{i}^{X}}{\tau _{k}^{Y}}$ and thus $E\left (\hat {\boldsymbol {f}}^{XY}\right )=E\left (\boldsymbol {f}^{X}\right )+E\left (\boldsymbol {f}^{Y}\right )$.

Consider now a perturbation of the form $f^{XY}_{(ik)}=\hat {f}^{XY}_{(ik)}+\varepsilon h_{(ik)}$, where *𝜖* is small and ***h*** obeys *h*_(*i*∙)_ = 0 and *h*_(∙*k*)_ = 0 to preserve the margins. Taylor expansion around *𝜖* = 0 yields

$$ E\left( \boldsymbol{f}^{XY}\right)=E\left( \boldsymbol{f}^{X}\right)+E\left( \boldsymbol{f}^{Y}\right)+\varepsilon\sum\limits_{ik}\hat{E}_{(ik)}h_{(ik)}+\frac{\varepsilon^{2}}{2}\sum\limits_{ikjl}\hat{E}_{(ik)(jl)}h_{(ik)}h_{(jl)}+0(\varepsilon^3) $$

where $\hat {E}_{(ik)}$ and $\hat {E}_{(ik)(jl)}$ are the corresponding derivatives (A.6) and (A.7) evaluated at $\hat {\boldsymbol {f}}^{XY}$. One has $\hat {E}_{(ik)}=-\ln \hat {\tau }^{XY}_{(ik)}-1=-\ln {\tau ^{X}_{i}} -\ln {\tau ^{Y}_{k}}-1$, and the contribution of this first order term is zero in view of the conditions imposed on ***h***. The contribution of the second order term is negative in view of the negative semi-definiteness of $\hat {E}_{(ik)(jl)}$ established in the proof of Theorem 9, and finally $E\left (\boldsymbol {f}^{XY}\right )\le E\left (\boldsymbol {f}^{X}\right )+E\left (\boldsymbol {f}^{Y}\right )+0(\varepsilon ^{3})=E\left (\hat {\boldsymbol {f}}^{XY}\right )+0(\varepsilon ^{3})$, showing $\hat {\boldsymbol {f}}^{XY}$ to be a local maximum of the effective entropy within the set of bivariate distributions with fixed margins. The latter set being convex, this local maximum is the global maximum in view of the concavity of *E*(***f***).

The above argument fails for the reduced entropy: its first-order derivative turns out to be $\hat {R}_{(ik)}=-\ln {b_{i}^{X}}-\ln {b_{k}^{Y}}-{\alpha _{i}^{X}}{\alpha _{k}^{Y}}$ where ${\alpha _{i}^{X}}={\sum }_{j} s_{ij}^{X}{f_{j}^{X}}/{b_{j}^{X}}$ and ${\alpha _{k}^{Y}}={\sum }_{l} s_{kl}^{Y}{f_{l}^{Y}}/{b_{l}^{Y}}$, and ***h*** can always be chosen so that $-\varepsilon {\sum }_{ik}{\alpha _{i}^{X}}{\alpha _{k}^{Y}}h_{ik}>0$, thus ruining subadditivity. □
